# A MALDI-TOF mass spectrometry-based method for detection of copy number variations in *BRCA1* and *BRCA2* genes

**DOI:** 10.3389/fmolb.2023.1301652

**Published:** 2024-01-11

**Authors:** Hongjun Zhou, Xin He, Jiadong Zhao, Zhu Mei, Xiayan Zhang, Wen Yuan, Hui Dong

**Affiliations:** ^1^ Nanjing Shenyou Institute of Genome Research, Nanjing, China; ^2^ Agena Bioscience (Shanghai) Co., Ltd., Shanghai, China; ^3^ Department of Gastroenterology, Shanghai General Hospital, Shanghai Jiao Tong University School of Medicine, Shanghai, China

**Keywords:** MALDI-TOF mass spectrometry, targeted sequencing, copy number variation, *BRCA1*, *BRCA2*

## Abstract

**Background:** Identifying germline mutations in *BRCA1* and *BRCA2* genes (*BRCAs*) would benefit the carriers in multiple aspects. In addition to single-nucleotide variations and small indels, copy number variations (CNVs) is also an indispensable component of identifiable mutations in *BRCAs*. A sensitive, rapid and throughput-flexible method to detect CNVs would be preferred to meet the rising clinical requirements for *BRCAs* testing.

**Methods:** We developed a MALDI-TOF-MS-based method (MS assay) which included three steps: first, multiplex end-point PCR followed by a single base extension reaction; second, automated analyte transfer and data acquisition; third, data analysis. We applied MS assay to detect CNVs in *BRCAs* in 293 Chinese patients with ovarian or pancreatic cancer. All the samples were examined by targeted next-generation sequencing (TS) simultaneously. Samples were further cross-validated by multiplex ligation-dependent probe amplification (MLPA) if the results from MS assay and TS were inconsistent. Long range PCR was then applied to identify the exact breakpoints in *BRCAs*.

**Results:** MS assay introduced highly multiplexed panels to detect CNVs of *BRCAs* semi-quantitatively. Simplified on-board data analysis was available for MS assay and no complex bioinformatics was needed. The turnaround time of MS assay was less than 8 hours with a hands-on time of only 40 min. Compared to TS, MS assay exhibited higher sensitivity (100% vs. 75%) and was more flexible in throughput, with the reagent cost per sample remaining constant no matter how many samples were examined per assay. A total of eight CNVs in *BRCAs* were detected from the 293 samples, and the molecular breakpoints were successfully identified in five samples through long-range PCR followed by Sanger sequencing.

**Conclusion:** Our results suggested that MS assay might be an effective method in primary screening for CNVs in genes such as *BRCAs*, especially when short turnaround time and/or high sensitivity is a top priority.

## Background

*BRCA1* and *BRCA2* genes are recognized as the primary inherited causes of breast and ovarian cancer since they were discovered in 1990s (Miki et al., 1994; Wooster et al., 1995). Germline mutations in *BRCA1* and *BRCA2* genes not only increase the risk of breast and ovarian cancer, but also contribute to the susceptibility of pancreatic and prostate cancer (Breast Cancer Linkage Consortium, 1999; Thompson et al., 2002). For carriers of *BRCA1* mutations, the risk of developing breast and ovarian cancer by age of 80 years is estimated to be 72% and 44% respectively, while it is 69% and 17% respectively for *BRCA2* carriers (Kuchenbaecker et al., 2017). Mutations in *BRCA2* also cause a 5–10% lifetime risk for pancreatic cancer, while *BRCA1* carriers might have two to four times risks compared to non-carriers (van Asperen et al., 2005; Ferrone et al., 2009; Mocci et al., 2013; Zhen et al., 2015; Roberts et al., 2016). In the case of prostate cancer, *BRCA1* and *BRCA2* mutations would lead to 8.6% and 15% cumulative risks respectively by age of 65 years (Thompson et al., 2002; Venkitaraman, 2002; Kote-Jarai et al., 2011; Leongamornlert et al., 2012). Identifying germline mutations in *BRCA1* and *BRCA2* genes would benefit the carriers by taking risk-reducing interventions before they get a cancer, as well as providing valuable information on the therapeutic application of Poly (ADP-ribose) polymerase inhibitors after a cancer occurs (Fong et al., 2009; Domchek et al., 2010).

Genetic testing for *BRCA1* and *BRCA2* mutation has now become a useful tool for both clinical and healthcare management. Multiple guidelines on genetic testing have been published emphasizing the necessity of identifying *BRCA* carriers for preventive and therapeutic purposes (US Preventive Services Task Force et al., 2019; Daly et al., 2020; Pujol et al., 2021). Although the majority of pathogenic variants identified in *BRCA1* and *BRCA2* are single-nucleotide variations (SNVs) and small insertions/deletions (indels), large genomic rearrangements (LGRs) accounts for up to 21% of all pathogenic variants, indicating that LGRs is an indispensable component of identifiable mutations (Judkins et al., 2012). The NCCN Guidelines also emphasized the need for comprehensive testing of all types of mutations including LGRs based on full length sequencing of *BRCA1* and *BRCA2* genes (Daly et al., 2021a). However, LGRs could not be detected by conventional PCR-based methods, and alternative methods such as long-range PCR, fluorescent *in situ* hybridization, comparative genomic hybridization, multiplex ligation-dependent probe amplification (MLPA) and targeted next-generation sequencing (TS) have been developed (Lambros et al., 2007; Walsh et al., 2010; Hernan et al., 2012; Stuppia et al., 2012; Biesma et al., 2015; Kwong et al., 2015). These approaches have contributed a lot in identifying LGRs, but they are either complicated, time-consuming or expensive, which hinders their applications in large scale screening. A simple, time-saving and throughput-flexible method to detect LGRs would be preferred to meet the rising clinical requirements for *BRCA* testing.

Matrix-assisted laser desorption/ionization time-of-flight mass spectrometry (MALDI-TOF-MS) is an outstanding platform for single nucleotide polymorphisms (SNPs) detection, featured by simple assay design, high throughput, high accuracy, excellent resolution, and short analysis times (Storm et al., 2003). In this study, we developed a MALDI-TOF-MS-based method (MS assay) for rapid detection of copy number variations (CNVs), which are due to LGRs, in *BRCA1* and *BRCA2* genes. The major advantage of this method is introducing highly multiplexed panels to detect CNVs of targeted genes semi-quantitatively, characterized by short turnaround time, flexible throughput and simplified on-board data analysis. Using this method, we examined CNVs in *BRCA1* and *BRCA2* in 293 Chinese patients with ovarian or pancreatic cancer. MS assay exhibited higher sensitivity compared to TS, suggesting that it might be an effective method in primary screening for CNVs, especially when shorter turnaround time and/or high sensitivity is a top priority.

## Materials and methods

### Samples

Leftover peripheral blood samples after routine clinical tests were collected from patients with ovarian cancer (*n* = 289) or pancreatic cancer (*n* = 4) in Shanghai General Hospital. Genomic DNA (gDNA) was extracted from blood using the QIAamp Blood Kit (Qiagen, Hilden, Germany) following the manufacturer’s instructions. The concentration of genomic DNA was measured by Qubit 3.0 fluorometer (Thermo Fisher Scientific, Waltham, MA) using dsDNA HS assay (Q32854). The study was performed in accordance with the Declaration of Helsinki and approved by the Ethics Committee of Shanghai General Hospital (No. 2022KY019). Requirement for informed consent was waived by the ethics committee.

### MS assay for CNVs detection

**FIGURE 1:**
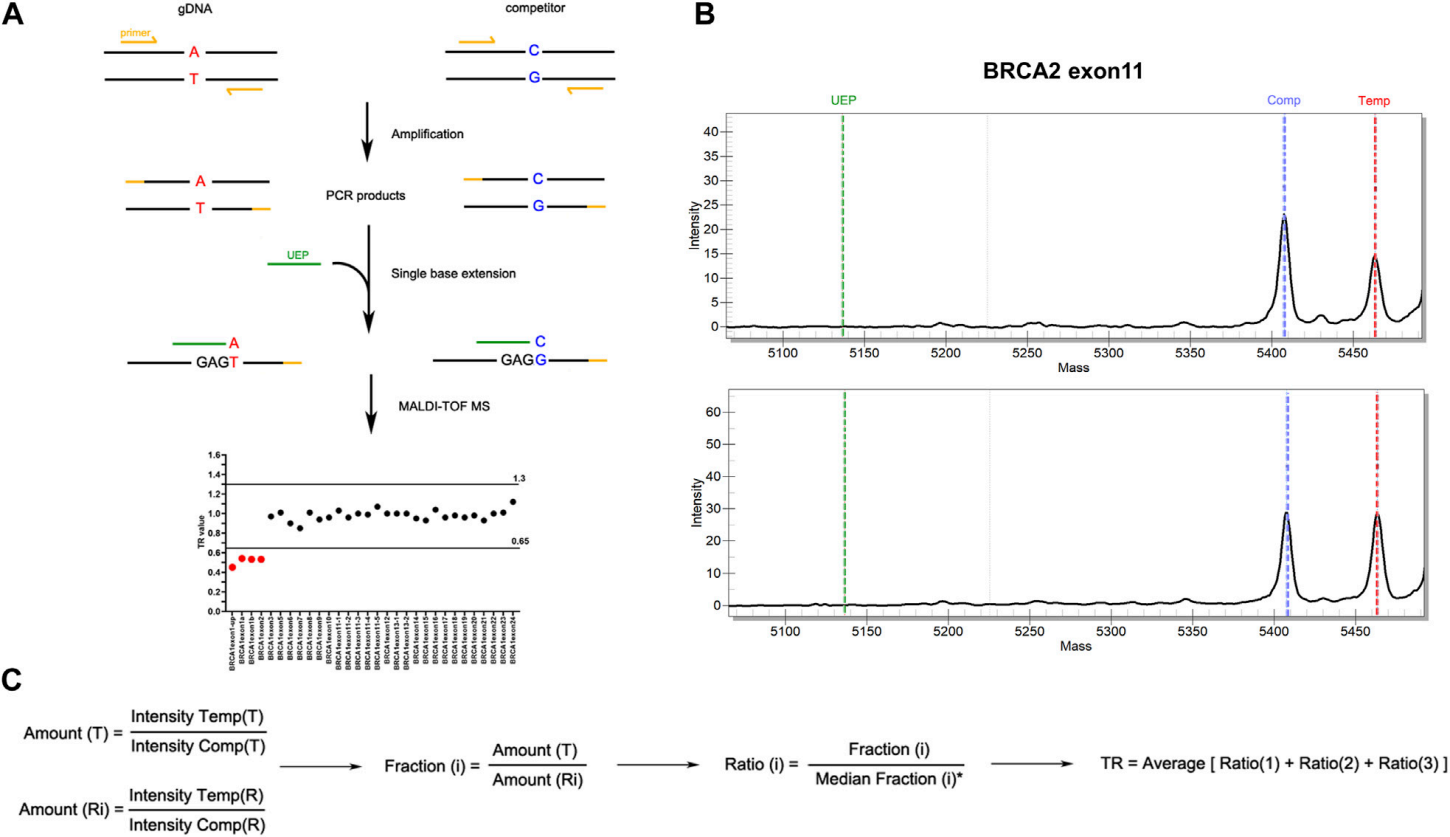
Workflow of MALDI-TOF-MS-based assay for CNV detection. **(A)** Experimental workflow of MALDI-TOF-MS-based assay. Multiplex PCR amplification and single base extension reactions were carried out with equal copies of gDNA and competitors inputted. The products were examined by MALDI-TOF-MS platform from which peak intensity was obtained. Target ratio (TR) value deriving from peak intensity of each fragment was then applied to evaluate CNVs. Fragments with TR ≤ 0.65 were considered to have deletions, while those with TR ≥ 1.30 were considered to have duplications. **(B)** Representative results of peak intensities of BRCA2 exon 11 fragments. The upper panel showed a CNV-positive sample with deletion in BRCA2 exon11, and the lower panel showed a wild type sample. **(C)** The algorithm for TR calculation. T, targeted gene fragments; R_i_, reference gene fragments (i = 1, 2, 3 which represent ALB, EIF2C1 and RNaseP respectively); Intensity Temp, peak intensity of template gDNA; Intensity Comp, peak intensity of competitor.

Three steps were included in MS assay: first, multiplex end-point PCR followed by a single base extension reaction, which took about 5.5 h with a hands-on time of 25 min; second, automated analyte transfer and data acquisition, which took about 100 min with a hands-on time of only 5 min; third, data analysis which took a hands-on time of 5–10 min. More specifically, each exon of *BRCA1* (NM_007294) and *BRCA2* (NM_000059) genes, as well as fragments of internal reference genes including *RNaseP*, *EIF2C1* and *ALB* genes, were amplified following by single base extension in multiplexed panels. Both primers for amplification and unextended primers (UEP) were designed using Assay Design Suite (version 2.2, Agena Bioscience). To be noted, primer sequences should not contain any SNPs with minor allele frequency >0.5%. Oligonucleotides that were only one base different from the corresponding gDNA fragments, namely competitors, were amplified and extended in parallel with gDNA using the same primers (Figure 1A). The sequences of primers for multi-plex PCR and UEP were listed in Supplementary Table S1.

All the exons of *BRCA1* and *BRCA2* were examined in a total of four multiplex PCR amplification and extension reactions which were carried out using iPLEX Pro Reagents Kit (Agena Bioscience) according to the manufacturer’s instructions. Briefly, equal copies of gDNA and competitors were inputted into one tube and PCR amplification was performed as followings: 30°C for 10 min followed by 96°C for 2 min, then 5 cycles of 96°C 45 s, 65°C 30 s and 72°C 1 min, 40 cycles of 96°C 45 s, 60°C 30 s and 72°C 1 min, with a final step of 72°C 5 min. PCR products were then treated with shrimp alkaline phosphatase, followed by a single-base extension which was performed as followings: 97°C 45 s, 97°C 5 s plus 5 repetitions of 52°C 5 s and 80°C 5 s for 40 cycles, and then 72°C 3 min. After extension, the products were desalted and spotted onto a SpectroCHIP (Agena Bioscience) using the MassARRAY Chip Prep Module (Agena Bioscience). SpectroCHIPs were scanned using the MassARRAY™ Analyzer 4 (Agena Bioscience) and the spectra were processed using the TYPER™ (Agena Bioscience) software to get the peak intensity for each allele. Target ratio (TR) value deriving from peak intensity of each fragment was applied to evaluate CNVs (Figure 1B). The algorithm for TR calculation was shown in Figure 1C. The cut-off of TR value was established using both CNV-positive standard samples purchased from the Coriell Institute (Camden, NJ) (Supplementary Table S2) and negative control samples which was proved to have diploid gene copy of *BRCA1* and *BRCA2* by MLPA. Fragments with TR ≤ 0.65 were considered to have deletions, while those with TR ≥ 1.30 were considered to have duplications. Batch calculation of TR value for multiple samples was performed in RStudio. The R code is available upon reasonable request.

### Hybrid capture-based targeted next-generation sequencing

The gDNA was fragmented and ligated to adaptors according to the protocol of NanoPrep™ DNA Library Preparation Kit (Nanodigmbio, Nanjing, China). Targeted fragments were captured by xGen^®^ Predesigned Gene Capture Pools (IDT, IA, United States) which covered all coding exons and flanking noncoding regions of *BRCA1* and *BRCA2* genes, and hybridization was carried out using xGen^®^ Hybridizaiton and Wash Kit and Universal Blockers (IDT, IA, United States). Then the DNA library was sequenced on MGISEQ-2000 instrument using MGISEQ-2000RS High-throughput Sequencing Kit (PE 150) (MGI, Shenzhen, China). Sequencing data was processed by GATK (https://gatk.broadinstitute.org/). Briefly, adaptor sequences in raw sequencing data were marked using MarkIlluminaAdapters, then aligned to human reference genome (version hg19) using BWA-MEM. Duplicated reads were identified using MarkDuplicates and base quality score recalibration was performed using BaseRecalibrator. Germline variants including point mutation and small indels were called by HaplotypeCaller, while germline CNVs was called by GermlineCNVCaller.

### MLPA assay for CNVs detection

CNVs in *BRCA1* and *BRCA2* genes were also examined using SALSA MLPA Probemix P002-D1 BRCA1 and P090-C1 BRCA2 (MRC-Holland, Amsterdam, Netherlands) respectively according to the manufacturer’s instructions. Genomic DNA was denatured at 98°C for 5 min, then the hybridization of probes to genomic DNA was performed at 95°C 1 min and 60°C 16 h. Hybridized probes were then ligated with a Ligase-65 master mix at 54°C 15 min and 98°C 5 min. PCR amplification of ligated probes was performed as followings: 35 cycles of 95°C 30 s, 60°C 30 s and 72°C 60 s, and then 72°C 20 min. Electrophoresis was performed on ABI 3500 (Thermo Fisher Scientific, Waltham, MA), and data analysis was done by Coffalyser.NET software (MRC-Holland, Amsterdam, Netherlands).

### Identification of breakpoints by long range PCR

Long range PCR was applied to explore and identify the exact breakpoints for samples with CNVs in *BRCA1* or *BRCA2* genes detected by MS-based assay. PCR was performed with TaKaRa LA Taq (Takara Bio, Otsu, Japan) in a final volume of 50 μL as followings: 94°C 1 min, 30 cycles of 98°C 10 s and 68°C 15 min, and then 72°C 10 min. PCR products were purified with the DiaSpin DNA Gel Extraction Kit (Diamond, Shanghai, China) and subjected to Sanger sequencing. Data analysis were performed by Sequencing Analysis v5.2 software (Thermo Fisher Scientific, Waltham, MA). Repetitive elements, such as LTR, LINE, Alu and MIR were identified by RepeatMasker and annotated through searching UCSC Genome Browser (http://genome.ucsc.edu).

## Results

### MS assay for detection of CNVs in *BRCA1* and *BRCA2* genes

A MS assay was developed for detection of CNVs in *BRCA1* (NG_005905.2) and *BRCA2* (NG_012772.3) genes, of which the turnaround time costed less than 8 hours, with a hands-on time of only 40 min. The throughput of MS assay was quite flexible, varying from one to more than a thousand samples per day, while the reagent cost per sample remained constant no matter how many samples were examined per assay. Besides, simplified on-board data analysis was available for MS assay and no complex bioinformatics was needed, which would greatly facilitate its application in clinical labs. More details of MS assay were described in Methods.

**FIGURE 2:**
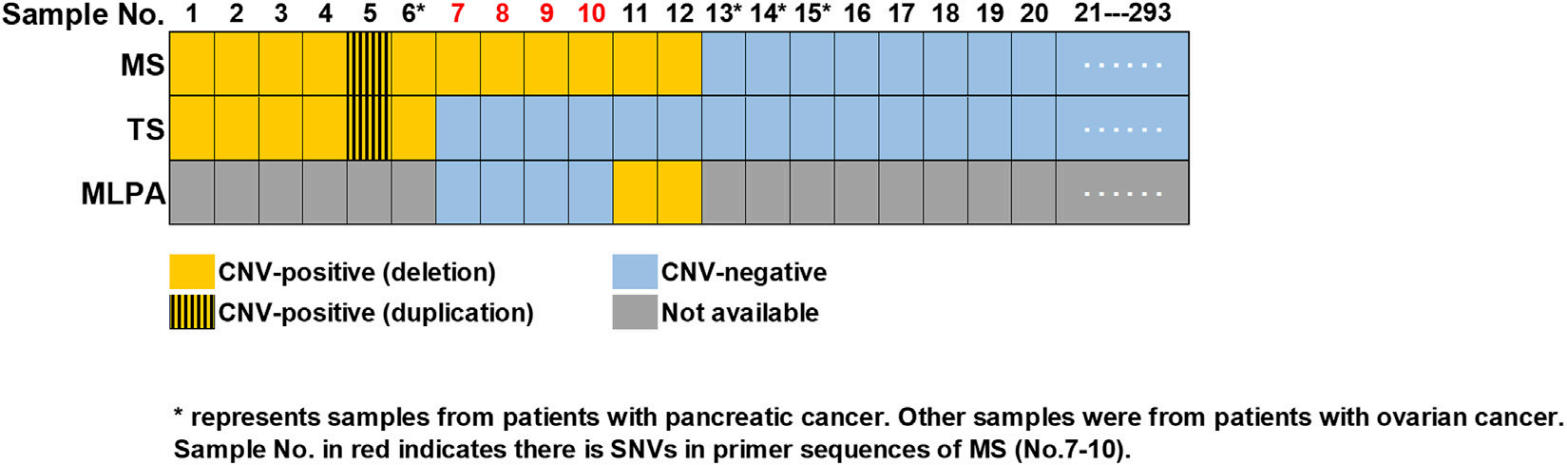
Summary of CNVs detected in 293 samples. Consistent results were obtained by MS assay and TS in 287 samples (No.1-6 and No.13-293), with six (No.1-6) being CNV-positive and 281 being CNV-negative (No.13-293). Sample No.7-10 were validated as CNV-negative, while sample No.11-12 were validated as CNV- positive by MLPA. Sample No. in red (No.7-10) indicated there were SNVs in the primer sequences of MS assay. “*” represented samples from patients with pancreatic cancer, while all the other samples were from patients with ovarian cancer. MS, MALDI-TOF-MS-based assay; TS, targeted sequencing; MLPA, multiplex ligation-dependent probe amplification.

Blood samples from 289 patients with ovarian cancer and four patients with pancreatic cancer were collected and subjected to CNVs detection through MS assay. The characteristics of enrolled patients were shown in Supplementary Table S3A. A total of 281 samples were found to be CNV-negative, while 11 patients with ovarian cancer (No. 1–5, 7–12) and one patient with pancreatic cancer (No. 6) were identified to carry CNVs in *BRCA1* or *BRCA2* genes by MS assay (Figure 2).

### Detection of CNVs in *BRCA1* and *BRCA2* genes by TS and MLPA

All the 293 samples mention above were examined by TS simultaneously. Through TS, a total of 47 unique pathogenic SNVs were identified in 56 individuals (Supplementary Table S3B). CNVs analysis showed that 287 samples (including No. 7–12) were CNV-negative, while five patients with ovarian cancer (No. 1–5) and one patient (No. 6) with pancreatic cancer were identified to carry CNVs in *BRCA1* or *BRCA2* genes (Figure 2). Thus, the results of six samples (No. 7–12) were inconsistent between TS and MS assay.

We then performed MLPA to validate whether there were CNVs or not in sample No. 7–12. Two samples (No. 11-12) were validated by MLPA as CNV-positive, while the other four (No. 7–10) were CNV-negative. Further analysis of the TS results indicated that in the cases of sample No. 7–10, there were SNVs in the amplification primer binding sites of MS assay (Figure 2; Supplementary Table S4), which might lead to false positive results in MS assay. Meanwhile, sample No. 11-12 that were CNV-positive identified by both MS assay and MLPA was recognized as CNV-negative in the TS sequencing results, indicating the results of TS were false negative.

### Characterization of CNVs in *BRCA1* and *BRCA2* genes

**FIGURE 3:**
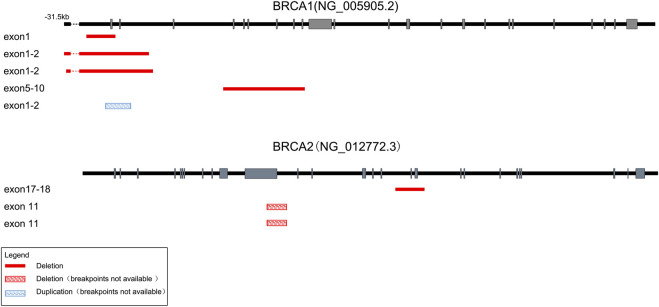
Schematic of the detected large genomic rearrangements in *BRCA1* and *BRCA2* genes. A total of eight CNVs were detected, among which seven were deletions (four in *BRCA1* and three in *BRCA2*), while only one was duplication in *BRCA1*. Deletions with identified breakpoints were depicted with red boxes, while deletions or duplications with unrecognized breakpoints were depicted with red or blue striped boxes respectively. The length of exons (grey bars) and introns (black lines) were not exactly to scale.

Totally, we detected CNVs in *BRCA1* and *BRCA2* genes in eight (2.7%) out of 293 samples. Consistent with previous reports (Sluiter and van Rensburg, 2011; Su et al., 2018), there were more deletions than duplications among the eight CNVs we identified. As shown in Figure 3 and Table 1, seven out of the eight CNVs were deletions (four in *BRCA1* and three in *BRCA2*), while only one CNV was duplication in *BRCA1*. Recurrent CNVs included deletions in exon 1-2 of *BRCA1* and in exon 11 of *BRCA2*. In fact, three samples were found to harbor CNVs in exon 1-2 of *BRCA1*, including two samples with deletions and one sample with duplication, suggesting that exon 1-2 of *BRCA1* might be a potential hotspot for CNV occurrence.

**TABLE 1:** The breakpoints and mechanisms of CNVs detected in this study.

Sample no.	CNVs	Size (bp)	Breakpoints	Mechanisms
*BRCA1*(NG_005905.2)				
1	exon 1-2 deletion	36934	g.61,101_98,034del	NAHR (ΨBRCA1/BRCA1)
2	exon 1 deletion	19857	g.73,379_93,235del	NHEJ (Non-Alu/AluSx)
3	exon 1-2 deletion	36934	g.61,422_98,355del	NAHR (ΨBRCA1/BRCA1)
5	exon 1-2 duplication	NA	NA	Unknown
6	exon 5-10 deletion	12117	g.110,371_122,487del	NAHR (AluSz6/AluJb)
*BRCA2*(NG_012772.3)				
4	exon 17-18 deletion	7892	g.48,409_56,300del	NAHR (AluSx/AluSz)
11	part of exon 11 deletion	NA	NA	Unknown
12	part of exon 11 deletion	NA	NA	Unknown

NA, not available; NAHR, non-allelic homologous recombination; NHEJ, non-homologous end-joining.

Moreover, we successfully identified the molecular breakpoints in five out of the eight CNV-positive samples by applying long-range PCR followed by Sanger sequencing, among which deletions of *BRCA1* exons 1-2 including g.61,242_98,355del and g.61,101_98,034del have been identified as recurrent CNVs in Chinese patients with breast cancer (Su et al., 2018). As shown in Table 1, four CNVs were caused by Alu-mediated or Ψ*BRCA1*-mediated non-allelic homologous recombination (NAHR), while another one was caused by non-homologous end-joining (NHEJ).

### Sensitivity and specificity of MS assay

To sum up, consistent results were obtained from 287 samples (No. 1–6 and No. 13–293) by both MS assay and TS, with six being CNV-positive and 281 being CNV-negative. Another six samples (No. 7–12) turned out to be CNV-negative in the result of TS, but CNV-positive in that of MS assay. Further validation by MLPA indicated that sample No. 11-12 were CNV-positive while sample No. 7–10 were CNV-negative. Thus, the sensitivity for MS assay and TS was 100% and 75%, while the specificity was 98.6% and 100% respectively (Table 2). MS assay showed higher sensitivity while the specificity was slightly lower when compared to that of TS.

**TABLE 2:** Sensitivity and specificity of MS assay and TS.

Methods	False positive	False negative	True positive	True negative	Sensitivity (%)	Specificity (%)
MS	n = 4	n = 0	n = 8	n = 281	100	98.6
TS	n = 0	n = 2	n = 6	n = 285	75	100

MS, MALDI-TOF-MS-based assay; TS, targeted sequencing.

## Discussion

It was suggested that unselected *BRCAs* genetic testing should be carried out in both ovarian and breast cancer patients regardless of family history and histopathology (Wu et al., 2017; Sun et al., 2022). Improvement in progression-free survival as well as in overall survival with maintenance Olaparib (a Poly (ADP-ribose) polymerase inhibitor) have been observed in patients with newly diagnosed advanced ovarian cancer and a *BRCA* variant (Moore et al., 2018; DiSilvestro et al., 2023). Furthermore, patients with HER2-negative early breast cancer and germline *BRCA1* or *BRCA2* variants could also benefit from adjuvant Olaparib treatment (Tutt et al., 2021). For non-cancer individuals, carriers of a *BRCA1* or *BRCA2* pathogenic variants was recommended to take more intensive screening and preventive strategies for breast, ovarian, prostate and pancreatic cancer (Elezaby et al., 2019; Daly et al., 2021b). The clinical demand for *BRCAs* testing keeps increasing, which calls for fast, throughput-flexible and cost-effective technologies to detect SNVs as well as CNVs. In this study, we developed the MS assay, which was featured by sensitive, time-saving and throughput-flexible, for CNV detection of *BRCA1* and *BRCA2*.

MALDI-TOF MS technology has been utilized for single nucleotide polymorphisms (SNPs) genotyping for two decades (Tost and Gut, 2002). In a recent study, the MS assay was also applied in CNV detection of *SMN1* and *SMN2* genes for spinal muscular atrophy genetic testing, showing highly concordant results with MLPA (Jin et al., 2022). In this study, we developed a MS assay for CNV detection of *BRCA1* and *BRCA2* genes. MLPA has also been widely applied in clinical labs for CNV detection of *BRCAs* (Lim et al., 2007; Lips et al., 2011). However, the experimental procedure of MLPA usually takes 2 days and the throughput is relatively low (45 testing samples plus three reference samples per batch), which might hinder its wide application in clinical practice. Although the throughput of TS is much higher, it is also time-consuming when compared to MS assay. The highly-automatized procedure of MS assay only takes less than 8 hours to generate results for 96 testing samples per batch, with a hands-on time of only 40 min. Besides, our results indicated that the sensitivity of MS assay is higher than TS (100% vs. 75%), implying that MS assay might be more effective in primary screening for CNV-carriers.

Among the 293 patients involved in this study, we identified a total of 64 carriers (21.8%) of pathogenic variants of *BRCA1* and *BRCA2* genes, including 56 patients (19.1%) carrying pathogenic SNVs/small indels, and eight patients (2.7%) carrying CNVs through TS and MS assay. Our results indicated that CNVs accounted for 12.5% (eight out of 64) of all pathogenic mutations in *BRCA1* and *BRCA2* genes, suggesting that examination of CNVs should also be carried out to comprehensively identify pathogenic mutation carriers of *BRCAs* in clinical practice. To be noted, mismatches in the primer binding sites of MS assay may lead to a false-positive result of deletion. In fact, we found four false-positive samples (sample No. 7–10) from the results of MS assay which were caused by SNVs in the primer binding sites as revealed by TS. Similarly, SNVs located on the MLPA probe binding sites have been observed to cause false positive results (Agaoglu et al., 2022). Another independent approach, such as TS, long-range PCR or fluorescent *in situ* hybridization is recommended to further validate the CNV-positive results derived from MS assay or MLPA.

It was reported more than 40% of the intronic sequences of *BRCA1* consist of Alu elements which were responsible for recombinational “hot spots”. Meanwhile, there were only 17% Alu sequences in *BRCA2*, which might explain why the incidence of rearrangements was lower in *BRCA2* than in *BRCA1* (Ewald et al., 2009). In addition, rearrangement involving Ψ*BRCA1* might also constitute a “hot spot” for recombination (Puget et al., 2002). Consistently, we observed Alu-mediated genomic rearrangements in both *BRCA1* and *BRCA2* in two out of the eight CNV-positive samples, and Ψ*BRCA*1-mediated rearrangement in another two samples. Two CNV-positive samples harbored *BRCA1* exon 1-2 deletion, which has been reported to be recurrent in Chinese patients with breast cancer (Su et al., 2018). Our results further suggested *BRCA1* exon 1-2 deletion might be a “hot spot” for recombination mediated by either Alu element or Ψ*BRCA1*.

The MS assay has several limitations. First, although three internal reference genes (*RNaseP*, *EIF2C1* and *ALB*) as well as a highly homologous competitor were included for double calibration of gene dosage in the MS assay, the quality of mass spectrometric signal might impact its accuracy for dosage quantification. Thus, the MS assay is recommended for detection of germline CNVs in high-quality samples, while it is not suitable for low-quality samples or samples with low tumor purity. Another limitation of MS assay is its lower specificity than TS, which is caused by mismatches in the primer binding sites and could not be completely avoided. In order to minimize potential mismatches, we excluded any SNPs with minor allele frequency >0.5% from primer sequences. However, as *BRCA1* and *BRCA2* variation is ethnic-specific, the minor allele frequency data are mainly derived from Caucasian populations of Europe and North America and might not be accurate in the case of Chinese population (Bhaskaran et al., 2019). Further improvement in primer design might be made by drawing information from Chinese-specific reference database such as db*BRCA*-Chinese (https://genemutation.fhs.um.edu.mo/dbbrca-chinese/). Besides, the specificity of MS assay would also be improved by simultaneously applying another primer set avoiding mismatches in the current set, which is now under development.

In summary, we developed a MALDI-TOF MS-based assay for CNV detection of *BRCA1* and *BRCA2* genes, which was characterized by high sensitivity, time-saving, and flexible throughput. Our results indicated that MS assay might be an effective method in primary screening for CNV-carriers, especially when short turnaround time and/or high sensitivity is a top priority. Application of MS assay is expected to satisfy the requirements of increasing demand for genetic testing of *BRCAs*, and could be easily expanded to the detection of CNVs in other genes in clinical practice and population screening.

## Data Availability

The raw data supporting the conclusion of this article will be made available by the authors, without undue reservation.

## References

[B1] Agaoglu N. B. Unal B. Akgun Dogan O. Zolfagharian P. Sharifli P. Karakurt A. (2022). Determining the accuracy of next generation sequencing based copy number variation analysis in Hereditary Breast and Ovarian Cancer. Expert Rev. Mol. Diagn 22, 239–246. 10.1080/14737159.2022.2048373 35240897

[B2] Bhaskaran S. P. Chandratre K. Gupta H. Zhang L. Wang X. Cui J. (2019). Germline variation in BRCA1/2 is highly ethnic-specific: evidence from over 30,000 Chinese hereditary breast and ovarian cancer patients. Int. J. Cancer 145, 962–973. 10.1002/ijc.32176 30702160 PMC6617753

[B3] Biesma H. D. Schouten P. C. Lacle M. M. Sanders J. Brugman W. Kerkhoven R. (2015). Copy number profiling by array comparative genomic hybridization identifies frequently occurring BRCA2-like male breast cancer. Genes Chromosom. Cancer 54, 734–744. 10.1002/gcc.22284 26355282

[B4] Breast Cancer Linkage Consortium (1999). Cancer risks in BRCA2 mutation carriers. J. Natl. Cancer Inst. 91, 1310–1316. 10.1093/jnci/91.15.1310 10433620

[B5] Daly M. B. Pal T. Berry M. P. Buys S. S. Dickson P. Domchek S. M. (2021a). Genetic/familial high-risk assessment: breast, ovarian, and pancreatic, version 2.2021, NCCN clinical practice guidelines in oncology. J. Natl. Compr. Canc Netw. 19, 77–102. 10.6004/jnccn.2021.0001 33406487

[B6] Daly M. B. Pal T. Berry M. P. Buys S. S. Dickson P. Domchek S. M. (2021b). Genetic/Familial high-risk assessment: breast, ovarian, and pancreatic, version 2.2021, NCCN clinical practice guidelines in oncology. J. Natl. Compr. Canc Netw. 19, 77–102. 10.6004/jnccn.2021.0001 33406487

[B7] Daly M. B. Pilarski R. Yurgelun M. B. Berry M. P. Buys S. S. Dickson P. (2020). NCCN guidelines insights: genetic/familial high-risk assessment: breast, ovarian, and pancreatic, version 1.2020. J. Natl. Compr. Canc Netw. 18, 380–391. 10.6004/jnccn.2020.0017 32259785

[B8] DiSilvestro P. Banerjee S. Colombo N. Scambia G. Kim B. G. Oaknin A. (2023). Overall survival with maintenance Olaparib at a 7-year follow-up in patients with newly diagnosed advanced ovarian cancer and a BRCA mutation: the SOLO1/GOG 3004 trial. J. Clin. Oncol. 41, 609–617. 10.1200/JCO.22.01549 36082969 PMC9870219

[B9] Domchek S. M. Friebel T. M. Singer C. F. Evans D. G. Lynch H. T. Isaacs C. (2010). Association of risk-reducing surgery in BRCA1 or BRCA2 mutation carriers with cancer risk and mortality. JAMA 304, 967–975. 10.1001/jama.2010.1237 20810374 PMC2948529

[B10] Elezaby M. Lees B. Maturen K. E. Barroilhet L. Wisinski K. B. Schrager S. (2019). BRCA mutation carriers: breast and ovarian cancer screening guidelines and imaging considerations. Radiology 291, 554–569. 10.1148/radiol.2019181814 31038410

[B11] Ewald I. P. Ribeiro P. L. Palmero E. I. Cossio S. L. Giugliani R. Ashton-Prolla P. (2009). Genomic rearrangements in BRCA1 and BRCA2: a literature review. Genet. Mol. Biol. 32, 437–446. 10.1590/S1415-47572009005000049 21637503 PMC3036053

[B12] Ferrone C. R. Levine D. A. Tang L. H. Allen P. J. Jarnagin W. Brennan M. F. (2009). BRCA germline mutations in Jewish patients with pancreatic adenocarcinoma. J. Clin. Oncol. 27, 433–438. 10.1200/JCO.2008.18.5546 19064968 PMC3657622

[B13] Fong P. C. Boss D. S. Yap T. A. Tutt A. Wu P. Mergui-Roelvink M. (2009). Inhibition of poly(ADP-ribose) polymerase in tumors from BRCA mutation carriers. N. Engl. J. Med. 361, 123–134. 10.1056/NEJMoa0900212 19553641

[B14] Hernan I. Borràs E. de Sousa Dias M. Gamundi M. J. Mañé B. Llort G. (2012). Detection of genomic variations in BRCA1 and BRCA2 genes by long-range PCR and next-generation sequencing. J. Mol. Diagn 14, 286–293. 10.1016/j.jmoldx.2012.01.013 22426013

[B15] Jin W. Yang Z. Tang X. Wang X. Huang Y. Hui C. (2022). Simultaneous quantification of SMN1 and SMN2 copy numbers by MALDI-TOF mass spectrometry for spinal muscular atrophy genetic testing. Clin. Chim. Acta 532, 45–52. 10.1016/j.cca.2022.05.017 35643151

[B16] Judkins T. Rosenthal E. Arnell C. Burbidge L. A. Geary W. Barrus T. (2012). Clinical significance of large rearrangements in BRCA1 and BRCA2. Cancer 118, 5210–5216. 10.1002/cncr.27556 22544547 PMC3532625

[B17] Kote-Jarai Z. Leongamornlert D. Saunders E. Tymrakiewicz M. Castro E. Mahmud N. (2011). BRCA2 is a moderate penetrance gene contributing to young-onset prostate cancer: implications for genetic testing in prostate cancer patients. Br. J. Cancer 105, 1230–1234. 10.1038/bjc.2011.383 21952622 PMC3208504

[B18] Kuchenbaecker K. B. Hopper J. L. Barnes D. R. Phillips K. A. Mooij T. M. Roos-Blom M. J. (2017). Risks of breast, ovarian, and contralateral breast cancer for BRCA1 and BRCA2 mutation carriers. JAMA 317, 2402–2416. 10.1001/jama.2017.7112 28632866

[B19] Kwong A. Chen J. Shin V. Y. Ho J. C. Law F. B. Au C. H. (2015). The importance of analysis of long-range rearrangement of BRCA1 and BRCA2 in genetic diagnosis of familial breast cancer. Cancer Genet. 208, 448–454. 10.1016/j.cancergen.2015.05.031 26271414

[B20] Lambros M. B. Natrajan R. Reis-Filho J. S. (2007). Chromogenic and fluorescent *in situ* hybridization in breast cancer. Hum. Pathol. 38, 1105–1122. 10.1016/j.humpath.2007.04.011 17640550

[B21] Leongamornlert D. Mahmud N. TymrakiewiczSaunders M. E. Dadaev T. Castro E. Goh C. (2012). Germline BRCA1 mutations increase prostate cancer risk. Br. J. Cancer 106, 1697–1701. 10.1038/bjc.2012.146 22516946 PMC3349179

[B22] Lim Y. K. Lau P. T. Ali A. B. Lee S. C. Wong J. E. Putti T. C. (2007). Identification of novel BRCA large genomic rearrangements in Singapore Asian breast and ovarian patients with cancer. Clin. Genet. 71, 331–342. 10.1111/j.1399-0004.2007.00773.x 17470134

[B23] Lips E. H. Laddach N. Savola S. P. Vollebergh M. A. Oonk A. M. Imholz A. L. (2011). Quantitative copy number analysis by Multiplex Ligation-dependent Probe Amplification (MLPA) of BRCA1-associated breast cancer regions identifies BRCAness. Breast Cancer Res. 13, R107. 10.1186/bcr3049 22032731 PMC3262220

[B24] Miki Y. Swensen J. Shattuck-Eidens D. Futreal P. A. Harshman K. Tavtigian S. (1994). A strong candidate for the breast and ovarian cancer susceptibility gene BRCA1. Science 266, 66–71. 10.1126/science.7545954 7545954

[B25] Mocci E. Milne R. L. Méndez-Villamil E. Y. Hopper J. L. John E. M. Andrulis I. L. (2013). Risk of pancreatic cancer in breast cancer families from the breast cancer family registry. Cancer Epidemiol. Biomarkers Prev. 22, 803–811. 10.1158/1055-9965.EPI-12-0195 23456555 PMC3739843

[B26] Moore K. Colombo N. Scambia G. Kim B. G. Oaknin A. Friedlander M. (2018). Maintenance Olaparib in patients with newly diagnosed advanced ovarian cancer. N. Engl. J. Med. 379, 2495–2505. 10.1056/NEJMoa1810858 30345884

[B27] Puget N. Gad S. Perrin-Vidoz L. Sinilnikova O. M. Stoppa-Lyonnet D. Lenoir G. M. (2002). Distinct BRCA1 rearrangements involving the BRCA1 pseudogene suggest the existence of a recombination hot spot. Am. J. Hum. Genet. 70, 858–865. 10.1086/339434 11880951 PMC379114

[B28] Pujol P. Barberis M. Beer P. Friedman E. Piulats J. M. Capoluongo E. D. (2021). Clinical practice guidelines for BRCA1 and BRCA2 genetic testing. Eur. J. Cancer 146, 30–47. 10.1016/j.ejca.2020.12.023 33578357

[B29] Roberts N. J. Norris A. L. Petersen G. M. Bondy M. L. Brand R. Gallinger S. (2016). Whole genome sequencing defines the genetic heterogeneity of familial pancreatic cancer. Cancer Discov. 6, 166–175. 10.1158/2159-8290.CD-15-0402 26658419 PMC4744563

[B30] Sluiter M. D. van Rensburg E. J. (2011). Large genomic rearrangements of the BRCA1 and BRCA2 genes: review of the literature and report of a novel BRCA1 mutation. Breast Cancer Res. Treat. 125, 325–349. 10.1007/s10549-010-0817-z 20232141

[B31] Storm N. Darnhofer-Patel B. van den Boom D. Rodi C. P. (2003). MALDI-TOF mass spectrometry-based SNP genotyping. Methods Mol. Biol. 212, 241–262. 10.1385/1-59259-327-5:241 12491915

[B32] Stuppia L. Antonucci I. Palka G. Gatta V. (2012). Use of the MLPA assay in the molecular diagnosis of gene copy number alterations in human genetic diseases. Int. J. Mol. Sci. 13, 3245–3276. 10.3390/ijms13033245 22489151 PMC3317712

[B33] Su L. Zhang J. Meng H. Ouyang T. Li J. Wang T. (2018). Prevalence of BRCA1/2 large genomic rearrangements in Chinese women with sporadic triple-negative or familial breast cancer. Clin. Genet. 94, 165–169. 10.1111/cge.13256 29582426

[B34] Sun L. Cui B. Wei X. Sadique Z. Yang L. Manchanda R. (2022). Cost-effectiveness of genetic testing for all women diagnosed with breast cancer in China. Cancers (Basel) 14, 1839. 10.3390/cancers14071839 35406611 PMC8997428

[B35] Thompson D. Easton D. F. Breast Cancer Linkage Consortium (2002). Cancer Incidence in BRCA1 mutation carriers. J. Natl. Cancer Inst. 94, 1358–1365. 10.1093/jnci/94.18.1358 12237281

[B36] Tost J. Gut I. G. (2002). Genotyping single nucleotide polymorphisms by mass spectrometry. Mass Spectrom. Rev. 21, 388–418. 10.1002/mas.1009 12666148

[B37] Tutt A. N. J. Garber J. E. Kaufman B. Viale G. Fumagalli D. Rastogi P. (2021). Adjuvant Olaparib for patients with BRCA1-or BRCA2-mutated breast cancer. N. Engl. J. Med. 384, 2394–2405. 10.1056/NEJMoa2105215 34081848 PMC9126186

[B38] US Preventive Services Task Force, Owens D. K. Davidson K. W. Krist A. H. Barry M. J. Cabana M. Caughey A. B. (2019). Risk assessment, genetic counseling, and genetic testing for BRCA-related cancer: US preventive services task force recommendation statement. JAMA 322, 652–665. 10.1001/jama.2019.10987 31429903

[B39] van Asperen C. J. Brohet R. M. Meijers-Heijboer E. J. Hoogerbrugge N. Verhoef S. Vasen H. F. (2005). Cancer risks in BRCA2 families: estimates for sites other than breast and ovary. J. Med. Genet. 42, 711–719. 10.1136/jmg.2004.028829 16141007 PMC1736136

[B40] Venkitaraman A. R. (2002). Cancer susceptibility and the functions of BRCA1 and BRCA2. Cell 108, 171–182. 10.1016/s0092-8674(02)00615-3 11832208

[B41] Walsh T. Lee M. K. Casadei S. Thornton A. M. Stray S. M. Pennil C. (2010). Detection of inherited mutations for breast and ovarian cancer using genomic capture and massively parallel sequencing. Proc. Natl. Acad. Sci. U. S. A. 107, 12629–12633. 10.1073/pnas.1007983107 20616022 PMC2906584

[B42] Wooster R. Bignell G. Lancaster J. Swift S. Seal S. Mangion J. (1995). Identification of the breast cancer susceptibility gene BRCA2. Nature 378, 789–792. 10.1038/378789a0 8524414

[B43] Wu X. Wu L. Kong B. Liu J. Yin R. Wen H. (2017). The first nationwide multicenter prevalence study of germline BRCA1 and BRCA2 mutations in Chinese ovarian cancer patients. Int. J. Gynecol. Cancer 27, 1650–1657. 10.1097/IGC.0000000000001065 28692638

[B44] Zhen D. B. Rabe K. G. Gallinger S. Syngal S. Schwartz A. G. Goggins M. G. (2015). BRCA1, BRCA2, PALB2, and CDKN2A mutations in familial pancreatic cancer: a PACGENE study. Genet. Med. 17, 569–577. 10.1038/gim.2014.153 25356972 PMC4439391

