# Supplementary material for: A MALDI-TOF mass spectrometry-based method for detection of copy number variations in BRCA1 and BRCA2 genes
Source: Front Mol Biosci. 2024 Jan 11;10:1301652. doi: 10.3389/fmolb.2023.1301652 (PMC10808477; doi:10.3389/fmolb.2023.1301652)
Supplement: Supplementary file 2 [file Table2.DOCX]

**Supplementary Table 2.** List of positive standard samples obtained from Coriell Institute.

| Samples | CNVs |
| --- | --- |
| NA18949 | BRCA1 exon 15-16 deletion |
| NA14626 | BRCA1 exon 13 duplication |
| NA02718 | whole gene of BRCA2 deletion |
| NA03330 | whole gene of BRCA2 duplication |
